# First Sequencing of Caprine Mdr1 (Abcb1) mRNA Due to Suspected Neurological Adverse Drug Reaction in a Thuringian Goat Following Extra-Label Use of Doramectin

**DOI:** 10.3389/fvets.2021.682393

**Published:** 2021-06-08

**Authors:** Daniela Nürnberger, Simon F. Müller, Melanie Hamann, Joachim Geyer

**Affiliations:** Faculty of Veterinary Medicine, Institute of Pharmacology and Toxicology, Justus Liebig University Giessen, Giessen, Germany

**Keywords:** MDR1, ABCB1, P-glycoprotein, goat, doramectin, drug sensitivity, neurological toxicity

## Abstract

The multidrug resistance gene MDR1 encodes for an efflux transporter called P-glycoprotein (P-gp). In the canine Mdr1 gene, a nonsense mutation was identified in certain dog breeds causing increased drug sensitivity to various P-gp substrates such as antiparasitic macrocyclic lactones. Symptoms of neurologic toxicity include ataxia, depression, salivation, tremor, apparent blindness, and mydriasis. In the current report, a Thuringian goat developed similar neurological signs after treatment with doramectin, a compound from the macrocyclic lactone class. Therefore, Mdr1 might be defective in this individual goat. For diagnostic purposes, sequencing of the complete mRNA transcript coding for caprine Mdr1 was performed to investigate a potential missense mutation. The Mdr1 transcripts of two related goats without drug sensitivity were also sequenced to allow differential diagnosis and were compared to the suspected drug-sensitive goat. The only position where the Mdr1 sequence from the suspected drug-sensitive goat differed was in the 3′-untranslated region, being a heterozygous single nucleotide polymorphism c.3875C>A. It can be suspected that this variant affects the expression level, stability, or translation efficiency of the Mdr1 mRNA transcript and therefore might be associated with the suspected drug sensitivity. To clarify this, further studies are needed, particularly investigating the Mdr1 mRNA and protein expression levels from brain material of affected goats. In conclusion, Mdr1 variants may exist not only in dogs, but also in individual goats. The current report provides the first Mdr1 mRNA transcript sequence of a goat and therefore represents the basis for more detailed Mdr1 sequence and expression analyses.

## Introduction

P-glycoprotein (P-gp) is an efflux transporter belonging to the family of ATP-binding cassette (ABC) transporters and is encoded by the multidrug resistance gene MDR1 (syn. ABCB1, ATP-binding cassette transporter subfamily B member 1) (1). In vertebrates, it is highly expressed in the blood–brain barrier and in various tissues such as the intestine, liver, and kidney. Here, P-gp restricts the entry of many drugs and xenobiotics into the central nervous system (CNS), limits their uptake from the gastrointestinal tract, and enhances the excretion via liver and kidney (2–4). The substrate spectrum of P-gp is broad and includes many compounds, e.g., anticancer agents, immunosuppressants, opioids, antiparasitic and antimicrobial drugs, cardiac drugs, steroid hormones, and many more (5–7). Antiparasitic drugs from the macrocyclic lactone class including ivermectin, doramectin, and moxidectin are also typical and high-affinity P-gp substrates (8–11). Drug sensitivities toward macrocyclic lactones were first described in the early 1980s and were mostly referred to Collie dogs that received ivermectin (12). Later the term “ivermectin-sensitive Collie” was used to describe this drug-sensitive phenotype (13, 14), which is characterized by ataxia, depression, excessive salivation, tremors, recumbency, apparent blindness, and mydriasis. The hypothesis of a genetic defect in the canine Mdr1 as a possible reason for this drug sensitivity was formulated some years later following observations in Mdr1 knockout laboratory mice. When these mice were treated with therapeutic doses of ivermectin for mite infection, severe neurological toxicity occurred while the Mdr1 wild-type mice were completely unaffected (15). Today, it is well-established that the increased drug sensitivity in Collies and related dog breeds is caused by nt230(del4) 4-bp gene deletion mutation (syn. ABCB1-1Δ) in the canine Mdr1 gene. This mutation leads to a premature stop codon and subsequently to a non-functional protein (16), resulting in an increased penetration of P-gp substrates into the CNS.

Interestingly, signs of intoxication seen in ivermectin-sensitive Collies are consistent with the neurological signs observed in a goat of the current report: a group of goats was treated subcutaneously with 0.3 mg/kg of a doramectin product approved for deworming in cattle and sheep. One of these goats, a 1-year-old female Thuringian goat (26 kg body weight), developed neurological signs at about 12 h after treatment. According to the animal owner, symptoms included ataxia, salivation, tremor, apathy, blindness, disorientation, mydriasis, and recumbency. The goat showed no water or food intake, swallowing reflex was absent, and rumen motility was decreased.

So far, no data are available from the literature describing neurological signs in goats after treatment with doramectin. The Federal Office of Consumer Protection and Food Safety (Germany) conducted a research in the European pharmacovigilance database following an official data request by the authors of this report. According to these search results, only a few cases of suspected adverse events have been reported in goats after administration of doramectin and ivermectin worldwide (search period from January 1, 2000 to December 31, 2020). However, considering these few reports on doramectin, the terms ataxia, lethargy, and apathy are mentioned frequently.

As the adverse reactions of these suspected drug-sensitive goats as well as those of the affected goat of the current case phenocopy quite well the ivermectin sensitivity of the Collie (with Mdr1 mutation), we hypothesized a putative Mdr1 mutation in these animals. Therefore, we performed diagnostic sequencing of the full-length Mdr1 transcript of the suspected drug-sensitive Thuringian goat and of two related (sire and half-sibling) non-sensitive goats to allow differential diagnosis.

## Materials and Methods

### Blood Samples

Blood samples were received for diagnostic Mdr1 sequencing from three Thuringian goats, among them, two 1-year-old females and one 2-year-old male. While one of the female goats developed neurological signs after treatment with doramectin and therefore was suspected to be drug-sensitive, the other two related animals (sire and half-sibling) did not show any adverse events after receiving the same doramectin dose and therefore were considered non-sensitive. The blood samples of the two non-sensitive related animals were also sent for diagnostic sequencing, because the owner wanted to be sure that they are not affected by a potential Mdr1 gene defect and to allow differential diagnosis. A total of 0.5 ml of anticoagulated blood was mixed with 1.3 ml of RNA*later* (Sigma-Aldrich, Taufkirchen, Germany) each and was further subjected to mRNA isolation.

### Sequencing of Caprine Mdr1

A predicted goat Mdr1 mRNA sequence (GenBank Accession Number XM_018047299.1) was used to design appropriate oligonucleotide primers for amplification and sequencing of the complete coding sequence (CDS). Therefore, eight overlapping fragments with a fragment size of about 600–1,100 bp were amplified with the following nucleotide primers: fragment 1, 5′-TCT TTC CGC TGG AGT CTG AAT C-3′ forward and 5′-GCG GGT GTT AAG TTC TCC AAC-3′ reverse; fragment 2, 5′-CGC TAT TCA AAT TGG CTT GAT AGG T-3′ forward and 5′-TTG TCC TCC AAA TGC AAT CAC AG-3′ reverse; fragment 3, 5′-AAT GGC GAC ATT CTT CAC CG-3′ forward and 5′-CTC ACC ACC CCG ATA ATC TC-3′ reverse; fragment 4, 5′-AAA CCA AGC ATC GAC AGC TAT TC-3′ forward and 5′-ACT TAG GGA TTC ACC AGG GGT-3′ reverse; fragment 5, 5′-GGA GAG TGA AGC GGT GGT T-3′ forward and 5′-ACA GCA AGC CTG GAA CCT AT-3′ reverse; fragment 6, 5′-GTA CCT GGT TTT CAG GTC GAT G-3′ forward and 5′-CTC AAT CTC CTC CTG GGA CAC-3′ reverse; fragment 7, 5′-ACT ACC CGA CTC GAC CAG AC-3′ forward and 5′-CTA GCG CTT TGC TCC AGC C-3′ reverse; fragment 8, 5′-GCT GGC ACA GTG TTT ATC GAT G-3′ forward and 5′-CTC TAC ATA GTT TCT CTG GTA GGT G-3′ reverse. All primers were ordered by Metabion (Planegg, Germany).

RNA isolation from RNA*later* blood was carried out using RiboPure-Blood Kit (Ambion/Life Technologies, Carlsbad, USA) and, subsequently, polyA^+^ mRNA was purified using Oligotex mRNA mini Kit (Qiagen, Hilden, Germany) according to the manufacturer's protocol. cDNA synthesis was then performed with SuperScript III First-Strand Synthesis System for RT-PCR (Invitrogen, Carlsbad, USA).

PCR amplification was performed with Phusion Flash High-Fidelity PCR Master Mix (ThermoFisher Scientific, Waltham, USA). Briefly, 2 μl of template cDNA were used in a final volume of 20 μl. A touchdown protocol was chosen consisting of an initial denaturation of 98°C for 2 min, followed by 10 loops of touchdown circle consisting of denaturation (98°C, 15 s), annealing (62–0.5°C per step, 15 s) and elongation (72°C, 35 s). After the touchdown phase, 30 cycles with denaturation temperature of 98°C for 15 s, annealing temperature of 57°C for 15 s and elongation for 35 s + 1 s per step (72°C) were performed, followed by final elongation at 72°C for 7 min.

PCR products were visualized on a 1.5% agarose gel before Hi Yield Gel/PCR DNA Fragment Extraction Kit (SLG, Gauting, Germany) was used for extraction and purification. Purified PCR products were sequenced by Microsynth Seqlab (Goettingen, Germany) with the same primers used for amplification. Received sequence data were analyzed and, subsequently, compared among each other and to the predicted caprine Mdr1 sequence using Finch TV 1.4 (Geospiza) and DNASTAR 16.0 software (Lasergene).

### Multiple Sequence Alignment

The amino acid sequences of the obtained caprine sequence and reference MDR1/Mdr1 sequences of various mammalian species were aligned by ClustalW algorithm in the DNASTAR 16.0 software. Sequences of sheep (NP_001009790.1), cattle (XP_024846789.1), horse (XP_014594657.1), dog (AAC02113.1), cat (NP_001164535.1), human (NP_000918.2), macaque (NP_001274251.1), camel (XP_031310691.1), alpaca (XP_015101231.1), pig (NP_001295175.1), mouse (isoform A: NP_035206.2 and isoform B: NP_035205.1), and rat (isoform A: AAS91649.1 and isoform B: NP_036755.3) were included for comparison. Protein sequence of goat was derived by selecting the common allele of all three goats, which was determined based on their known relationships. Visualization of amino acid sequences was performed with BOXSHADE software 3.21. The phylogenetic tree was created by uncorrected pairwise distance in DNASTAR and visualized by FigTree v.1.4.4 software.

## Results

### Diagnostic Mdr1 Sequencing Results

When a herd of six goats was treated with the antiparasitic drug doramectin at 0.3 mg/kg subcutaneously, one individual developed severe neurological signs characterized by ataxia, depression, excessive salivation, tremor, apparent blindness, and mydriasis about 12 h after drug application. To clarify if a hitherto unknown missense mutation in the Mdr1 gene might be responsible for this suspected drug sensitivity, the complete Mdr1 CDS of this goat was amplified and sequenced from blood-derived mRNA. For comparison, two closely related goats (sire and half-sibling) were sampled, which did not show any adverse drug reaction after doramectin application. At the time of analysis, there was only a predicted caprine Mdr1 mRNA sequence available (GenBank Accession No. XM_018047299.1) that was derived by Gnomon prediction from sequenced genome fragments of a San Clemente goat (GenBank Accession No. NC_030811.1). This sequence is further referred to as SC-goat Mdr1 sequence. The Mdr1 cDNA was amplified and sequenced in eight overlapping fragments and revealed a full-length CDS of 3855 bp, which is coding for the caprine 1284 amino acid P-gp. Obtained sequence data were submitted to the GenBank database with Accession No. MW365935. This sequence is further referred to as T-goat Mdr1 sequence.

**Figure 1:**
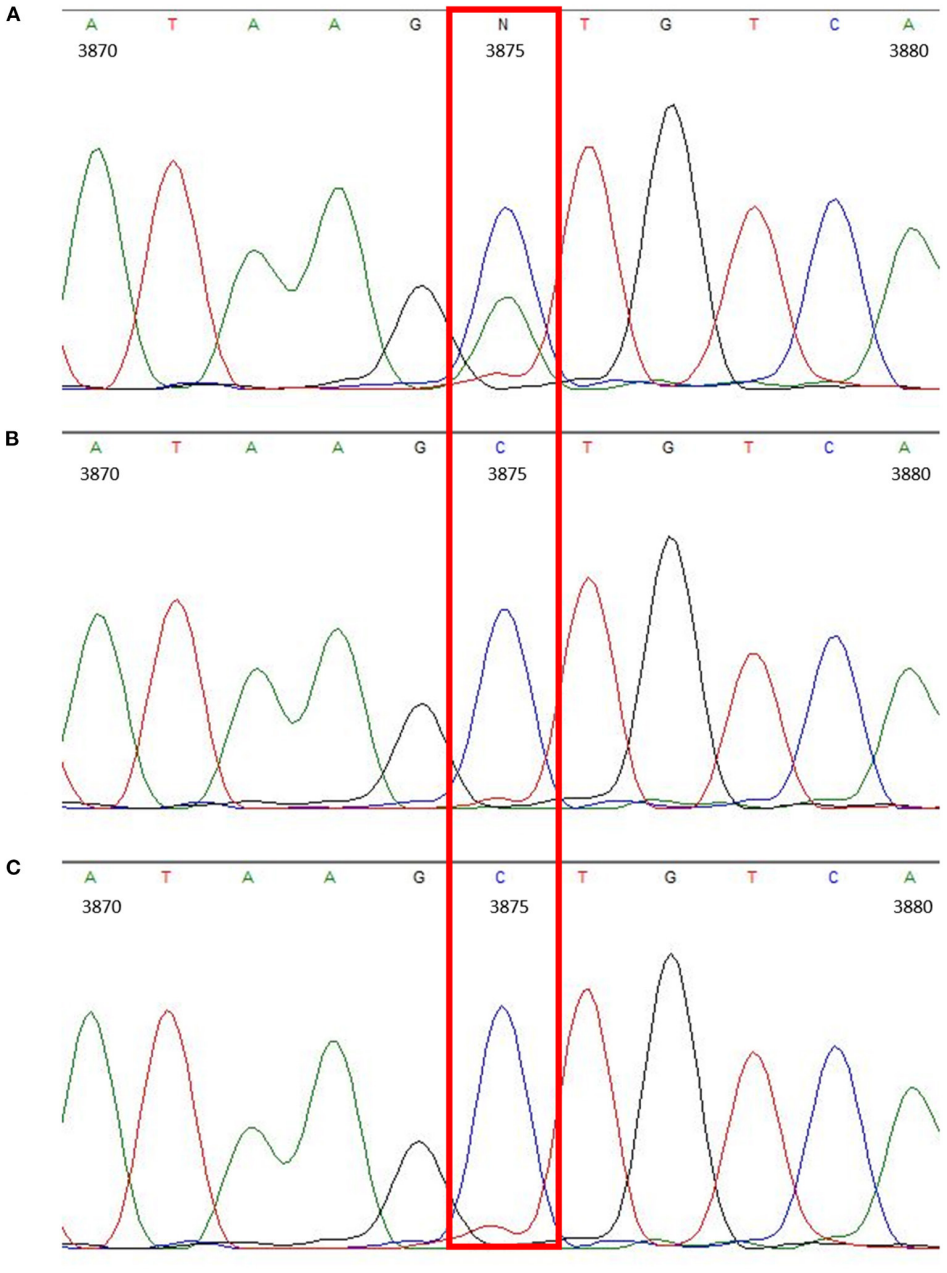
Chromatogram showing the heterozygous SNP located in the 3′-UTR (c.3875C>A) of the suspected drug-sensitive goat **(A)**. In comparison, chromatograms of the sire **(B)** and half-sibling **(C)**, which did not develop any neurological signs after treatment with doramectin.

Overall, the obtained sequences of all three Thuringian goats had a high level of identity. When checking the amplified Mdr1 sequences for differences, only one nucleotide position could be identified where the sequence of the suspected drug-sensitive goat differed from the two others, being a single nucleotide polymorphism (SNP) located in the 3′-untranslated region (3′-UTR) at position 3875 (C>A). The suspected drug-sensitive goat was heterozygous for the 3875 A allele (Figure 1). Although some further sequence variations were detectable among the three Thuringian goat sequences, these aberrant findings generally occurred either in only one of the non-sensitive animals, or in the suspected drug-sensitive goat as well as in one of the non-sensitive animals (Table 1). Therefore, these sequence variations did not allow to discriminate between the suspected drug-sensitive and the non-sensitive phenotype.

**Table 1:** Comparison of Mdr1 cDNA of the Thuringian goats with the SC-goat Mdr1 sequence.

**No**.	SNP^*^	**Amino acid change**	**Suspected drug-sensitive goat**	**Non-sensitive goat (sire)**	**Non-sensitive goat (half-sibling)**
1	371G>C	Cysteine to serine	C/C	C/C	C/C
2	507T>C	–	C/C	C/C	C/C
3	644G>A	Arginine to lysine	A/A	A/A	A/A
4	1014A>G	–	G/G	G/G	G/G
5	1017C>A	–	A/A	A/A	A/A
6	1206T>A	–	A/A	A/A	A/A
7	1374T>C	–	C/C	C/C	C/C
8	1632T>C	–	C/C	C/C	C/C
9	1833C>T	–	T/T	T/T	T/T
10	2355A>G	–	G/G	G/G	G/G
11	2559T>C	–	**C/C**	**C/C**	**T/C**
12	2715G>A	–	**A/A**	**A/A**	**G/A**
13	3489C>T	–	C/T	C/T	C/T
14	3525A>G	–	A/G	A/G	A/G
15	3576T>G	–	**T/G**	**T/G**	**T/T**
16	3580G>A	Alanine to threonine	**G/G**	**G/A**	**G/G**
17	3811A>G	Isoleucine to valine	**A/G**	**A/G**	**A/A**
18	3843A>G	–	A/G	A/G	A/G
19	3844G>A	Alanine to threonine	G/A	G/A	G/A
20	3′-UTR 3875C>A	–	**C/A**	**C/C**	**C/C**

*Positions where the three amplified Thuringian goat sequences differ among each other are indicated in bold*.

* *Compared to the predicted SC-goat Mdr1 sequence*.

Compared to the predicted SC-goat Mdr1 sequence, the three T-goat Mdr1 transcripts differed in several positions, all being SNPs (Table 1). Eight of these aberrant findings were homozygous and synonymous SNPs. Two further variants were homozygous and non-synonymous SNPs, leading to an amino acid change from cysteine to serine (c.371G>C) or arginine to lysine (c.644G>A). Four sequence variations were heterozygous SNPs (c.3489C>T; c.3525A>G; c.3843A>G; c.3844G>A) from which only the last one was non-synonymous and results in an amino acid change from alanine to threonine. All these SNPs occurred in all Thuringian goats compared to the SC-goat Mdr1 sequence and, therefore, can potentially be regarded as breed specific.

### Multi-Species Sequence Alignment

**Figure 2:**
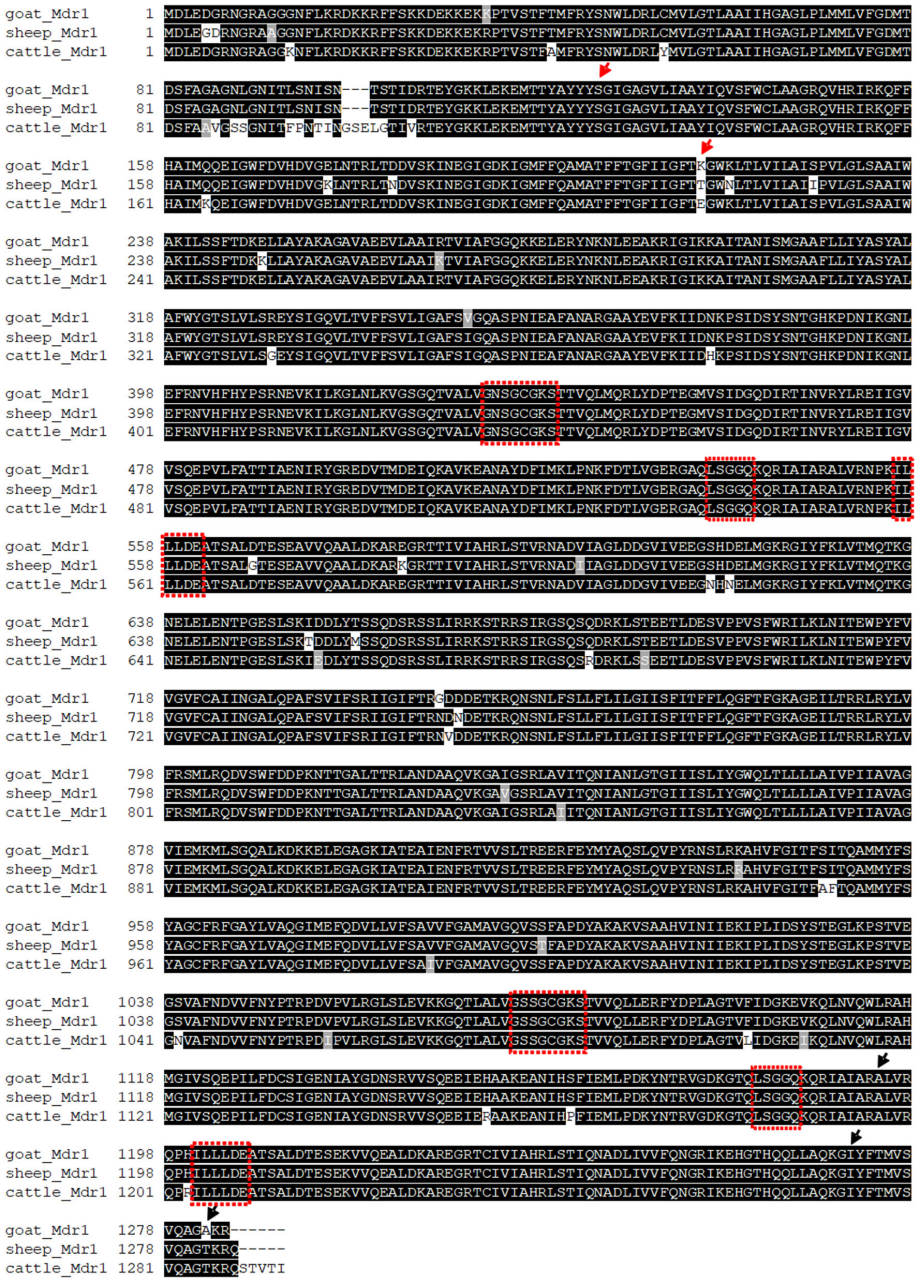
Alignment of amino acid sequences of the determined T-goat Mdr1 sequence and reference sequences of sheep and cattle with conserved Walker A motif, Walker B motif, and C motif (red boxes), which are characteristic for ABC-transporters. Arrows mark the positions of homozygote (red) and heterozygote (black) amino acid changes of the experimentally determined T-goat sequence compared to the predicted SC-goat Mdr1 sequence.

**Figure 3:**
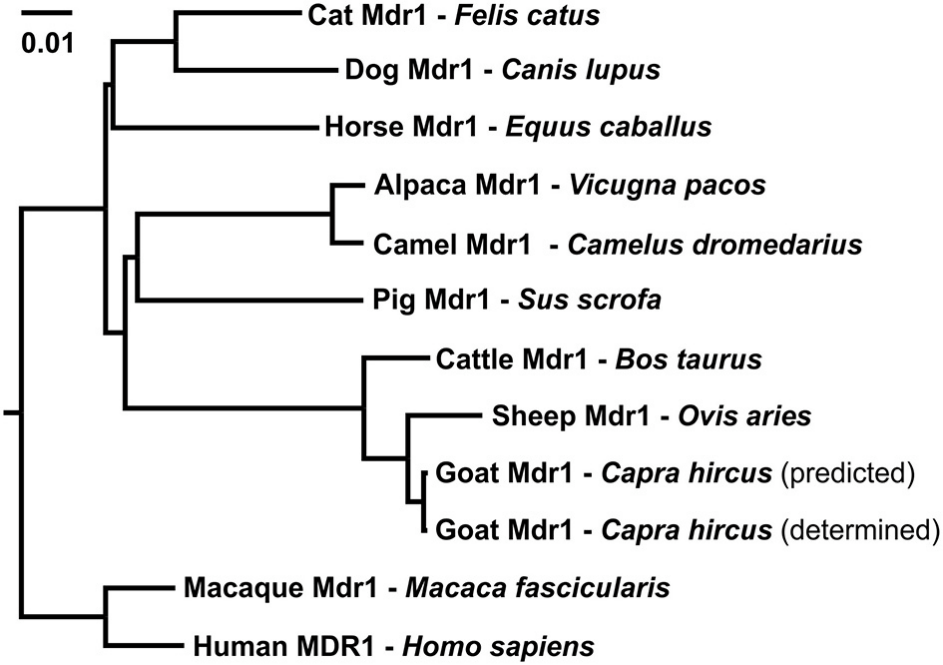
Phylogenetic tree of amino acid sequence relationships of Mdr1 from cat, dog, horse, pig, alpaca, camel, sheep, goat, cattle, macaque, and MDR1 of human. For goat Mdr1, the predicted SC-goat Mdr1 protein sequence as well as the experimentally determined T-goat Mdr1 sequence were included. The length of the scale bar represents 0.01 amino acid changes per site for horizontal distance.

Multiple sequence alignment of amino acid sequences was performed between the obtained Mdr1 goat protein sequence and the reference Mdr1 sequences of sheep, cattle, horse, dog, cat, human, macaque, camel, alpaca, pig, mouse, and rat (with isoform A and B of both rodent species). P-gp of sheep (98%) and cattle (97%) showed the highest percentage of identity, followed by horse, cat, human, macaque, camel, and alpaca (89%). Alignment showed 88% identity for dog and pig, 86% for rat and mouse (isoform A) and the lowest percentage of identity for isoform B of rat and mouse (79%). In particular, the Walker A motif, Walker B motif, and C motif, which are characteristic for ABC-transporters, are highly conserved and are shown for the three ruminant species in Figure 2. A phylogenetic tree is shown in Figure 3.

## Discussion

Mutations and polymorphisms in the MDR1/Mdr1 gene have been reported in humans (17), mice (18), dogs (16), and cats (19). In humans, numerous MDR1 SNPs have been identified, but there is no clear consensus regarding their functional and/or clinical consequence (20). In mice, drug sensitivity has been shown in Mdr1 knockout mice against ivermectin (15, 21). The same applies to dogs, in which the nt230(del4) 4-bp deletion mutation in the Mdr1 gene dramatically increases drug sensitivity to macrocyclic lactones, such as ivermectin, doramectin, or moxidectin (7, 11, 16). Typical symptoms of neurological toxicity induced by macrocyclic lactones in Mdr1 mutant dogs include ataxia, CNS depression, excessive salivation, tremor, apparent blindness, and even death.

In the current report, a Thuringian goat showed similar neurological signs after subcutaneous injection of 0.3 mg/kg doramectin as well. To date, neither any report about drug sensitivity to doramectin in goats, nor any mutations in the Mdr1 gene of goats have been published. However, a research in the European pharmacovigilance database that was conducted by the Federal Office of Consumer Protection and Food Safety (Germany) revealed global occurrence of few cases of suspected adverse events in goats following the administration of ivermectin or doramectin. In these cases of adverse event reports after administration of doramectin, the following terms are commonly mentioned: ataxia, lethargy, and apathy. The adverse drug reactions of these suspected drug-sensitive goats phenocopy quite well the drug sensitivity of Mdr1 mutant dogs against macrocyclic lactones (7). Based on this, it was hypothesized that the suspected doramectin-sensitive Thuringian goat of the present report might have defective expression or function of the Mdr1 drug efflux transporter in the blood–brain barrier. Therefore, we focused on searching for missense mutations or sequence variations in the mRNA coding sequence and flanking 3′- and 5′-UTR regions. However, it was not possible to analyze Mdr1 mRNA and protein expression at the blood–brain barrier as this would have required sampling from the brain. The Mdr1 transcript was PCR amplified and sequenced from the suspected drug-sensitive Thuringian goat as well as from two closely related animals (sire and half-sibling), which also received the doramectin treatment, but did not show any adverse events. The only sequence variation that could explain the different phenotypes was a heterozygous SNP in the 3′-UTR of the Mdr1 transcript, being c.3875C>A. It can only be speculated that this SNP might have affected Mdr1 expression that finally would have resulted in defective of reduced Mdr1 expression at the blood–brain barrier. This can, however, not be evidenced in the current case and needs further investigation.

A variety of possibilities exists, how alterations of 3′-UTRs can affect protein expression levels, i.e., changes of mRNA stability, changes in poly-A-tailing motifs, accessory-protein binding, as well as changes of microRNA binding sites. To address this question, we *in silico* screened the c.3875C>A sequence variation for potential changes of binding motives with various algorithms (RBPDB: http://rbpdb.ccbr.utoronto.ca and Scan for Motifs: http://crispr.otago.ac.nz/sfm/sfm_main.pl) but could not find any plausible motif. This might be due to the fact that available databases are mainly human specific or pan-mammalian, so their usefulness for goat-UTRs is limited and may yield false-negative results. To underline the possible impact of a 3′-UTR mutation, we want to hint to the recent work of Martinez et al. (22). They showed that CYP2B11 3′-UTR sequence variations, which are frequently present in dog breeds such as the Greyhound, can lead to decreased CYP2B11 protein abundance. To verify if there might be a similar causality for caprine Mdr1 expression for specific goat breeds or goats in general, an elaborate prospective study like the one mentioned above would be needed, to provide sufficient genetic and especially tissue samples to be able to prove or reject this hypothesis. Therefore, it finally remains unclear whether or not the c.3875C>A SNP located in the 3′-UTR impacted P-gp expression of the suspected drug-sensitive goat and thus is responsible for the observed differences in drug sensitivity. Furthermore, other mutations in the non-coding region or epigenetic modifications altering the Mdr1 gene expression cannot be excluded. Here, for example, promotor region mutations would also be possible. In the current case, however, a severe defect of the promotor seems to be unlikely since sequencing revealed amplification of both alleles. In total, further research with a larger number of affected animals and additional sample acquisition for measuring P-gp expression, especially in the brain, would be necessary in the future to verify a potential association of the c.3875C>A mutation with increased drug sensitivity to avermectins in the goat.

In the present case, besides pharmacogenetic and epigenetic causes, other reasons responsible for the neurological signs of the suspected drug-sensitive goat must be considered as well. According to the animal owner, neurological signs included ataxia, apathy, tremor, salivation, mydriasis, and recumbency, although the goat was able to stand and walk on its own when the animal was placed in an upright position. Obviously, the goat was blind and disorientated; moreover, swallowing reflex was absent and rumen motility was decreased. Of note, these symptoms occurred in close time with the doramectin treatment of the goat. Symptomatic treatment included subcutaneous infusions of saline (WDT) and Amynin (Merial) solutions, injections of Konstigmin 2.5 mg/ml (Vetoquinol) and Vitamin-B-Komplex pro inj. (Serumwerk), as well as Vetalgin 500 mg/ml (MSD) for several days. Additionally, the goat received ColoSan (SaluVet) and Pansenstimulans (WDT) orally when swallowing reflex was recovered. Symptoms lasted for about 1 week and then improved gradually. In total, symptoms and time course of intoxication are very similar to ivermectin and doramectin intoxications reported for Mdr1 mutant dogs (23, 24). However, there are reports of dogs treated with macrocyclic lactones that showed similar clinical symptoms but did not have the nt230(del4) Mdr1 mutation (25–27). Some of these cases include accidental ingestion and overdosing. In the current case, a doramectin product approved for cattle and sheep was administered to goats, implying an extra-label use. According to the product information, the manufacturer's dose recommendation for cattle and sheep is 0.2 mg/kg doramectin for intramuscular administration (accessed March 2021). This dose has also been administered to goats in some pharmacokinetic, milk kinetic, and efficacy studies with a subcutaneous route of administration (28–33). In the current case, 0.3 mg/kg doramectin were administered subcutaneously. However, this dose was well-tolerated by several other goats of the group treated with doramectin at the same time. Nevertheless, it is well-known that inter-individual factors, such as percent of body fat, age, diet, or disease, have an influence on the pharmacokinetics or pharmacodynamics of a drug and may therefore lead to variations in drug response (34). It should also be mentioned that the Thuringian goat has recovered almost completely, but according to the owner, blindness lasted for several months, which seems to be unusual for an intoxication with macrocyclic lactone drugs (25, 27). Therefore, other possible reasons for the occurrence of neurological adverse events should be considered. A variety of differential diagnoses exist for neurological disorders in goats, such as polioencephalomalacia/cerebrocortical necrosis, listeriosis, hepatic encephalopathy, further metabolic diseases, as well as many others. Blindness often occurs as a secondary finding in some of these diseases. Poisonings with toxic plants or chemicals may also lead to neurological signs associated with blindness (35, 36). In this regard, it should be noted that the goat was found on a surrounding pasture after doramectin treatment, which had been limed the day before.

In conclusion, this report provides the first sequence of the caprine Mdr1 based on blood-derived mRNA. Diagnostic Mdr1 sequencing revealed no missense mutations in the Mdr1 coding sequence of the goat showing neurological signs after treatment with doramectin, but we identified a heterozygous variation located in the 3′-UTR (c.3875C>A) where the sequence of the suspected drug-sensitive goat differed from the two related non-sensitive goats that were treated with doramectin as well. It finally remains unclear if this mutation is responsible for the observed differences in potential drug sensitivities among the three goats after treatment with doramectin. In addition, it is unknown if other mutations in the non-coding region or epigenetic mechanisms exist that may have altered Mdr1 gene expression in the affected goat. In this case, however, besides pharmacogenetic and epigenetic causes, other reasons responsible for the neurological signs cannot be excluded.

## Data Availability Statement

The raw data supporting the conclusions of this article will be made available by the authors, without undue reservation.

## Ethics Statement

Ethical review and approval was not required for the animal study because blood samples were received for diagnostic sequencing of the Mdr1 transcript due to suspected Mdr1-related drug sensitivity. For this reason, ethics approval was not necessary in agreement with the institutional animal welfare officer of the Justus Liebig University Giessen. Written informed consent was obtained from the animal owner for publication of the data. Written informed consent was obtained from the owners for the participation of their animals in this study.

## Author Contributions

DN, SM, MH, and JG conceived the diagnostic sequencing, analyzed and interpreted the data, and critically edited and revised the manuscript. DN performed the sequencing and drafted the first manuscript. DN and SM prepared figures and tables. All authors contributed to the article and approved the final version of the manuscript.

## Conflict of Interest

The authors declare that the research was conducted in the absence of any commercial or financial relationships that could be construed as a potential conflict of interest.

## Abbreviations

3′-UTR: 3′-untranslated region
5′-UTR: 5′-untranslated region
ABC: ATP-binding cassette
ABCB1: ATP-binding cassette transporter subfamily B member 1
CDS: coding sequence
CNS: central nervous system
MDR: multidrug resistance
P-gp: P-glycoprotein
SC-goat: San Clemente goat (predicted Mdr1 sequence)
SNP: single nucleotide polymorphism
T-goat: Thuringian goat (experimentally determined Mdr1 sequence).
